# Human Cervix Chip: A Preclinical Model for Studying the Role of the Cervical Mucosa and Microbiome in Female Reproductive Health

**DOI:** 10.1002/bies.70014

**Published:** 2025-05-22

**Authors:** Zohreh Izadifar, Anna Stejskalova, Aakanksha Gulati, Ola Gutzeit, Donald E. Ingber

**Affiliations:** ^1^ Urology Department Boston Children's Hospital and Department of Surgery, Harvard Medical School Boston Massachusetts USA; ^2^ Wyss Institute for Biologically Inspired Engineering Harvard University Boston Massachusetts USA; ^3^ IVF Unit, Department of Obstetrics and Gynecology Rambam Medical Center Haifa Israel; ^4^ Clinical Research Institute at Rambam (CRIR) Rambam Health Care Campus Haifa Israel; ^5^ Vascular Biology Program, Boston Children's Hospital and Department of Surgery Harvard Medical School Boston Massachusetts USA; ^6^ Harvard John A. Paulson School of Engineering and Applied Sciences Cambridge Massachusetts USA

**Keywords:** Cervix Chip, fertility, human in vitro model, microbiome, microfluidic, mucosal immunity, pathogen, pregnancy, pre‐term birth

## Abstract

Advancements in women's reproductive health have been hindered by insufficient knowledge and the underrepresentation of women in research, leading to symptom‐focused care with poor outcomes. Modeling female reproductive biology and disease pathophysiology has been challenging due to the complexity and dynamic nature of the female organs. Here, we briefly review recent advancements made with a new in vitro microfluidic organ‐on‐a‐chip model of the human cervix (Cervix Chip) that faithfully mimics key features of the cervix, including mucus production and physiological responses to hormonal, environmental, and microbial stimuli. We also discuss how this preclinical platform can provide a way to obtain unique insights into the role of mucosal immunity, genetic and risk factors, as well as microbiome and pathogen interactions in human cervix health and disease, while bridging knowledge gaps in fertility and pregnancy‐related conditions. By enabling preclinical drug screening and accelerating translational research, the Cervix Chip holds the potential to improve the development of therapeutics, diagnostics, and ultimately, the sexual and reproductive health of millions of women globally.

## Graphical abstract

The microfluidic Cervix Chip is an in vitro model of the human cervix lined by cervical epithelium interfaced with stromal cells cultured under dynamic flow that represents a new preclinical platform for studying mucosal immunity, host‐microbiome interactions, infections, fertility, and pregnancy health.

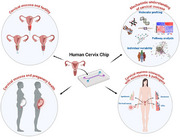

## 1 Introduction

Women comprise nearly half of the global population, and projections suggest that by 2050, over 65% of the world's countries will have a majority‐female population [1]. Despite this, women's health has been routinely overlooked in medical research. This neglect has not only led women to experience 25% more of their lives in poor health compared to men but has also resulted in an excess of $20 billion in annual healthcare costs related to female reproductive and sexual health [2]. As the demographic landscape shifts over the next three decades, the impact of the women's health gap is likely to magnify. Increased participation of women in all societal roles makes their health status an issue with far‐reaching consequences, underscoring the urgent need to bridge the disparity in women's healthcare.

The unique reproductive biology of women, characterized by organ‐specific physiology and functions, and the resultant systemic hormonal variations, is central to their overall health. These dynamics play a vital role not only in reproduction but also in influencing other bodily systems, including the brain [3, 4], gut [5, 6], bones [7], and cardiovascular system [8]. These intricacies of female reproductive biology and the systemic effects of related hormonal changes make this field challenging, perpetuating a persistent knowledge gap. As a result, clinical care often focuses on symptom management and treatments that frequently fail or lead to relapses and complications. To revolutionize healthcare for women, it is essential to address this challenge by improving our understanding of both physiological and pathological processes relevant to women's health and diseases. This will empower researchers to identify new therapeutic targets for effective treatments applicable to a wide range of female reproductive and sexual conditions.

Traditionally, animal models and conventional cell cultures have been the gold standard of biomedical research. However, their applicability to human health, particularly within the context of female biology, is limited. Take, for example, the fact that female mice do not menstruate and human‐derived bacterial species fail to engraft in animals [9, 10]. Culture of human cervical and vaginal cells on conventional two‐dimensional (2D) dishes, Transwell inserts, and microspheres have been used to approach this problem, but they lack key physiological characteristics of the cervicovaginal mucosa and its physical microenvironment [11, 12]. Even advanced human cell culture models, such as organoids, fall short because they lack the structural and functional complexities of the human reproductive organs, including tissue‐tissue interfaces and dynamic fluid flow, and thus, they fail to faithfully replicate many of their responses [13, 14, 15, 16]. To truly enable the mechanistic study of women's sexual and reproductive conditions, we must develop in vitro models that accurately simulate the physiology and pathophysiology of the human female reproductive tract.

**FIGURE 1:**
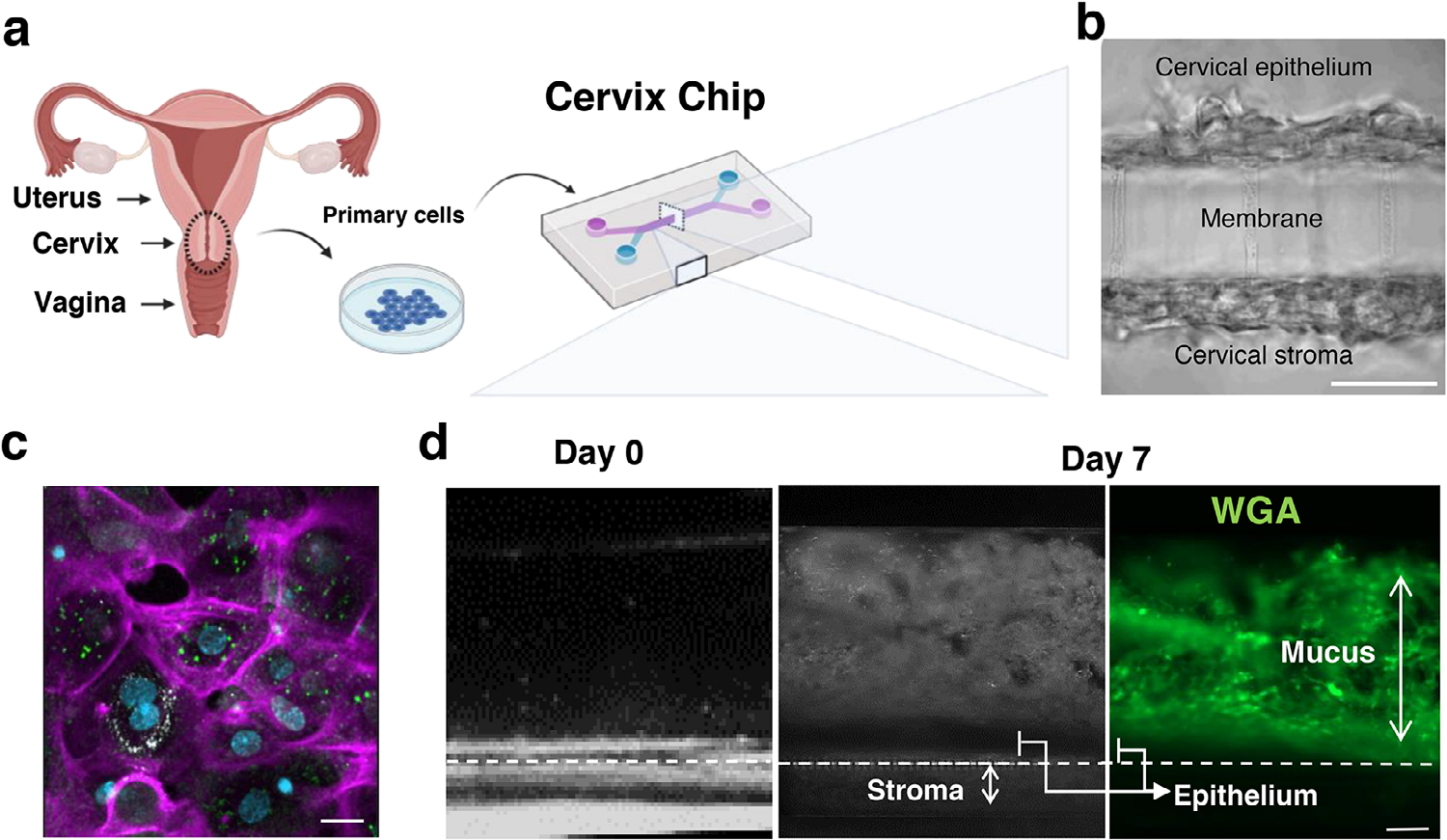
The microfluidic human Cervix Chip model. (a) Schematic of the organs of the upper and lower female reproductive tract showing the cervix at the interface between the uterus above and vagina below (left), as well as how cells isolated from the cervix are cultured within a microfluidic Organ Chip device to create the Cervix Chip (right). (b) Phase contrast micrographs of vertical cross‐sections of the Cervix Chip that is lined by primary cervical epithelial cells cultured in the top microfluidic channel on the surface of a porous membrane with cervical stromal cells cultured on the opposite side of the same membrane in the lower channel (bar, 50 µm). (c) Immunofluorescence micrograph of the epithelium within the Cervix Chip viewed from above showing expression of epithelium differentiation markers including MUC5B (green), β‐tubulin (white), and F‐actin (magenta), as well as nuclei stain (DAPI, blue) (bar, 25 µm). (d) Dark field (left, middle) and fluorescence (right) micrographs of vertical side views of the chips showing accumulation of a thick mucus layer above the epithelium on‐chip from day 0 to day 7 of culture, which appears as a white fuzz in dark‐field and as a green staining due to binding of fluorescently‐labeled wheat germ agglutinin (WGA), as shown in the right view (bar, 200 µm; dashed line indicates porous membrane).

A key female reproductive organ is the human cervix, which is located at the junction of the upper and lower reproductive tracts and connects the vagina to the uterus (Figure 1a). The cervix serves as the primary mucus‐producing organ of the reproductive system, and its mucosal secretions are essential for female sexual and reproductive health. They form a protective mucosal barrier along the entire length of the lower reproductive tract that enables stable microbiome cohabitation [17, 18], regulates sperm passage during conception [19], and protects the uterus and fetus from invading pathogens during pregnancy [20, 21]. Various health issues, including pregnancy complications, viral and bacterial infections, cervical cancer, and chronic pelvic conditions arise or worsen due to pathological changes in the cervical mucosa. However, the underlying mechanisms and processes of these conditions remain poorly understood because of limitations in current model systems. Moreover, it is not possible to study human host‐microbiome interactions in conventional static 2D or organoid cultures because the microbes rapidly overgrow the human cells, and this ‟contamination″ leads to cell injury and culture death.

Organ‐on‐Chip (Organ Chip) microfluidic culture technology has enabled an entirely new way to model the complex structure and function of human tissues and organs in vitro by integrating design principles of microengineering and cell biology. Human Organ Chips are able to recreate human organ‐ and tissue‐level structures and functions with in vivo‐like fidelity by providing cells with biomimetic mechanical, chemical, and microenvironmental cues [22]. Because the tissue experiences dynamic fluid flow, complex human microbiome also can be cultured in direct contact with human epithelium for extended times (multiple days) in vitro [23, 24]. This technology can be applied to help advance female sexual and reproductive health by enabling development of preclinical and physiologically relevant in vitro models of human reproductive tissues and organs. Here we briefly review recent advances made with an Organ Chip model of the human cervix [12] and discuss how this model can be leveraged to advance the field by providing a research platform for preclinical modeling and mechanistic study of the cervical mucosa and its role in sexual and reproductive health and diseases.

### 1.1 Cervix Chip: A High‐Fidelity In Vitro Model of the Human Cervix

We recently reported a new in vitro model of the human cervix that was developed using Organ Chip microfluidic culture technology [12]. This model uses a commercially available perfusable microfluidic chip (Emulate Inc., Boston, MA) containing two parallel microchannels separated by a thin porous membrane coated with an extracellular matrix. Primary cells obtained from human cervical epithelium and stroma are cultured in the different channels on opposite sides of the same membrane to recreate the epithelial‐stromal interface, while fluids are perfused independently through the top and bottom channels to mimic flow in the cervical lumen as well as interstitial flow of nutrient medium driven by vascular perfusion (Figure 1b). The human Cervix Chip was extensively characterized using a range of different functional, chemical, molecular, and cellular assays and shown to much more closely mimic the structure, function, and physiology of the human cervix in vivo [12] when compared to commonly used 2D and 3D culture systems [25, 26].

One of the major advantages of the human Cervix Chip is that it not only forms an epithelial barrier and expresses cellular markers of differentiated cervical epithelium (Figure 1c), but it also abundantly produces mucus that forms a visually observable protective layer on the surface of the epithelium with biochemical and hormone‐responsive properties similar to those observed in mucus within the human cervix in vivo (Figure 1d). To our knowledge, this has not previously been achieved with other in vitro cervix models, even in the most recently reported models using microfluidic culture technology [27, 28]. The Cervix Chip mucus was found to have the composition and biomolecular complexity seen in clinical samples of cervical mucus, including multiple mucin types (i.e., MUC5B, MUC4) [29] as well as their heterogenous composition of different glycosylation states (i.e., sialylated, sialofucosylated, and fucosylated O‐ and N‐glycans). Although direct comparison of the mucus volume produced from chip to that of cervix in vivo is not straightforward (due to significant scale differences and additional secretions from vagina in vivo), we were able to detect presence of both soluble mucins in the chip epithelium effluents as well as adherent physical mucus layer on‐chip similar to cervix in vivo. Leveraging the ability to control flow rates and patterns in the microfluidic chip, we also discovered the influence of micromechanical (flow‐dependent) cues on the formation of the two different types of cervical epithelium (endocervical and ectocervical) each with its distinct pattern of gene expression, innate immune response, and mucus glycosylation profile [12]. These results demonstrated that the Cervix Chip platform opens a new avenue for studying the biology of cervical epithelial and stroma cells, as well as mucus production, in a tissue‐ and organ‐relevant context, as well as their contributions to women's reproductive health and diseases.

**FIGURE 2:**
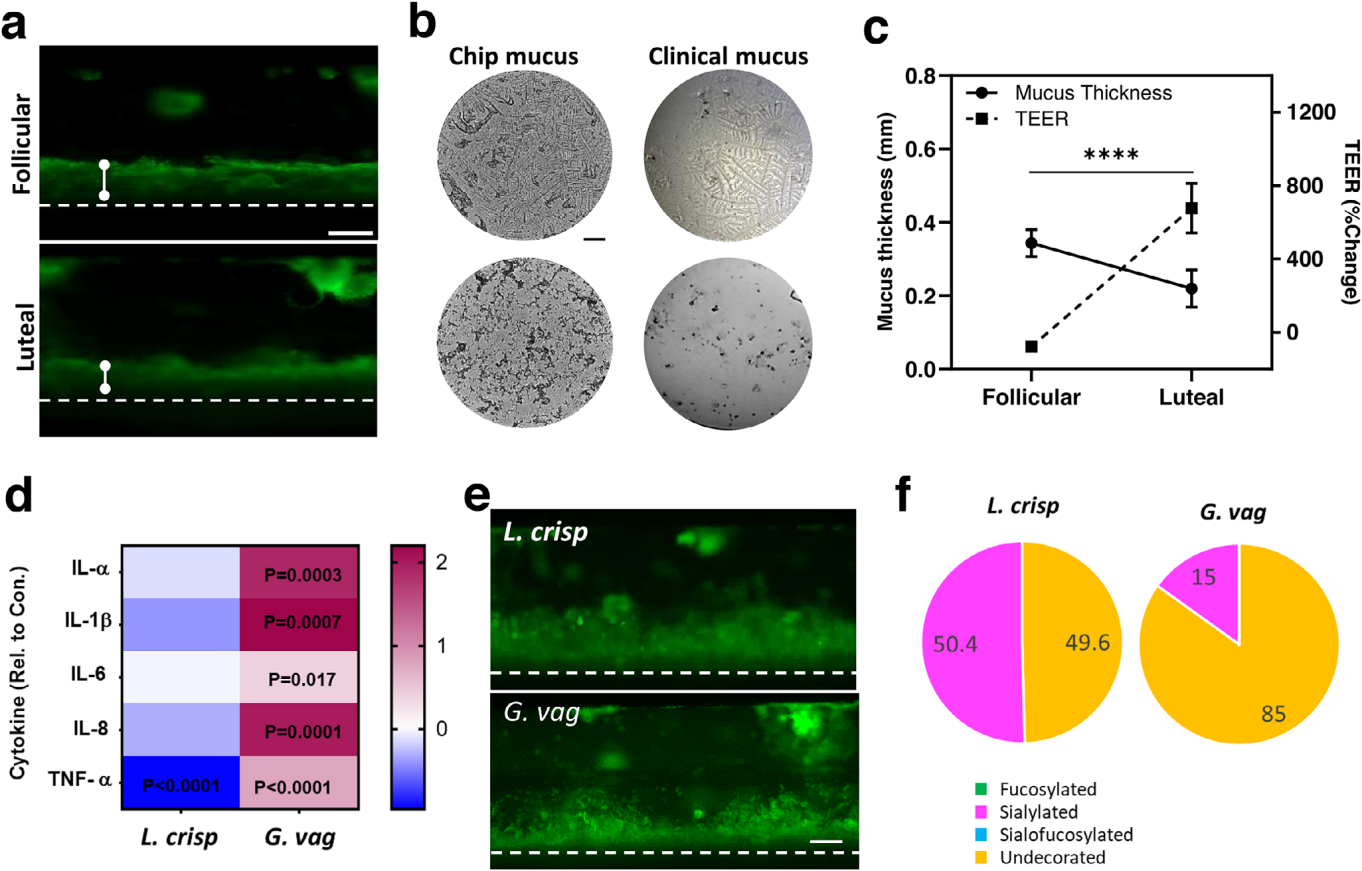
Physiological responses to sex hormones and bacterial co‐culture recapitulated in the human Cervix Chip. (a) Fluorescence micrographs of vertical side views of the chips cultured with follicular (high estrogen) and luteal (high progesterone) hormone levels when stained with WGA to visualize the mucus layer (thickness indicated by arrows; bar, 200 µm). (b) Bright field images of the Fern Tests obtained with mucus samples from the human Cervix Chip (left) and clinical human samples (right) during exposure to ovulatory (follicular, top) and non‐ovulatory (luteal, bottom) hormone levels (clinical mucus data reproduced with permission from [126]; bar, 200 µm). (c) Quantitative analysis of changes in the mucus thickness and barrier function measured by quantifying transepithelial electrical resistance (TEER) when the human cervical tissues were exposed to follicular and luteal hormone levels on‐chip. (d) Heat map showing Cervix Chip innate immune responses to co‐culture with *L. crispatus* (*L. crisp*) or dysbiotic *G. vaginalis* (*G. vag*), as indicated by changes in the protein levels of 5 relevant proinflammatory cytokines relative to controls (data shown represent Log_10_ fold change over control chip without bacteria). (e) Fluorescence micrographs of vertical side views of the chips cultured with *L. crisp* or *G. vag* and stained with WGA (green) (bar, 200 µm). (f) Pie charts representing the relative abundances of glycan types in the Cervix Chip mucus after 3 days of co‐culture with healthy (*L. crispatus*) versus dysbiotic (*G. vaginalis*) microbiome, as determined by glycomics analysis.

Sex hormones and their fluctuations throughout a woman's life have a significant impact on female physiology and disease states [30, 31, 32]; however, current models only provide limited information about the cell and molecular basis of these responses (e.g., gene and protein expression patterns). The Cervix Chip faithfully modelled physiological modulation of the cervical mucosa including, for example, cell‐ and tissue‐level functional responses to menstrual cycle hormones [12]. These studies revealed that the Cervix Chip produces a significantly thicker mucus layer with a high abundance of mucins and decorated glycosylations, highly branched mucus ferns, and a more permeable epithelial barrier when exposed to hormones associated with the follicular phase of the cycle compared to the luteal phase (Figure 2a–c). Importantly, these in vitro results obtained on‐chip correspond well to changes observed in human clinical samples. Changes in tissue barrier function that are known to occur during the transition from the follicular to the luteal phase were also recapitulated on‐chip. Future studies can further expand on these experiments to more precisely mimic cyclic hormonal changes that occur in vivo. Importantly, previously reported microfluidic culture models of the human cervix [27, 33] do not provide this level of physiological responses, clinical mimicry, or experimental versatility.

Another significant finding from studies in the Cervix Chip was that the human cervical tissues could be co‐cultured with living microbes for extended times, which enabled comprehensive analysis of host‐bacterial interactions. As opposed to previously reported in vitro models where bacterial overgrowth limits co‐culture studies to under 24 h, the Cervix Chip enabled 3 days of co‐culture, which we have extend even longer in recent studies (up to 5 days; unpublished observation). Replication of physiological conditions, for example by using intermittent fluid flow (similar to cervicovaginal discharge) and stringently minimizing use of non‐human sourced medium components, helped to enable this advancement. Chip cultures potentially can be carried out using a reduced starting inoculum load of bacteria and culturing them under anaerobic conditions to obtain even longer co‐culture times. Co‐culture under anaerobic conditions and reducing the size of the starting inoculum on‐chip can also be tested for further extension of the co‐culture time. This is important because the cervicovaginal microbiome is increasingly recognized as one of the important players in the health and disease of the female reproductive tract. Despite its importance, the mechanisms that underlie these host‐microbiome interactions still remain understudied. Advanced metagenomic and bacterial culture technologies have improved our understanding of which bacteria colonize the reproductive tract in symbiosis and dysbiosis states [34, 35]; however, absence of relevant and robust human in vitro models have made it difficult to obtain further insight into how these interactions occur. We demonstrated that the Cervix Chip reliably models mucosal responses to clinical bacterial isolates from healthy women and those with bacterial vaginosis. Co‐culture of these bacteria on‐chip within the epithelial lumen allowed longitudinal and end‐point analysis of live and fixed chips revealing multiple aspects of host‐microbiome interactions within the human cervix. This includes quantitative and/or qualitative information on bacteria colonization, epithelium injury (cell death and barrier disruption), effects of the optimal and non‐optimal (dysbiotic) microbiome on secretion of pro‐ and anti‐inflammatory cytokines, modulation of living mucus structure and composition (i.e., mucin glycosylation), and changes in secretory protein profiles (Figure 2d–f). The Cervix Chip model not only demonstrated its potential as a robust platform for basic and translational research, but also showcased how versatile characterization technologies can be integrated to better understand lower reproductive tract health and disease at the molecular, cell, tissue, and clinically relevant levels. We have also previously reported development of a preclinical Organ Chip in vitro model of human vagina (Vagina Chip), which showed similar robustness in terms of recapitulating vaginal mucosa structure, function, and physiological responses to hormones and co‐culture with vaginal microbiome [24]. Recently, by transferring mucus produced by a Cervix Chip to a Vagina Chip, we demonstrated that cervical mucus significantly reduces inflammation and epithelial damage caused by a dysbiotic microbiome commonly associated with bacterial vaginosis [36].

### 1.2 Improving Understanding of Human Cervical Mucosal Immunity

The human cervical mucosa and its physical and biochemical constituents of the overlying mucus layer it produces create a highly restrictive barrier at the entry of the uterus to protect the upper reproductive tract while also enhancing defense mechanisms in the lower reproductive tract. Clinical studies have made great advances in revealing the importance of mucosal immunity in risks associated with diseases or adverse health outcomes [37, 38, 39, 40]; however, still little is known about how host factors and associated mechanisms modulate health and disease in the female reproductive tract. In fact, the field has been in desperate need of well‐designed studies and model systems that can provide a better understanding of host mucosal immunity and how it is modulated by internal and external stimuli, such as genetics, hormones, environmental factors, and microbiome.

One of the primary unknowns in this field is the full composition and function of various proteins, lipids, glycans, and other secretory molecules that are produced by the epithelium, found in the mucus layer, or present within the stroma or surrounding microenvironment. Identifying these components can provide a way to further investigate the mechanisms and processes involved in their production, regulation, and most importantly, contribution to tissue homeostasis and mucosal immunity to microbial invasion. With the significant advances in molecular analysis techniques, such as mass spectrometry‐based multi‐omics [41, 42] (i.e., proteomics, metabolomics, glycomics), the human cervix and its mucus layer can be comprehensively studied to identify their composition with greater detail than ever before. One main challenge with performing these studies using clinical samples is that it is extremely difficult to delineate and identify the exact processes or mechanisms involved due to contributions of many unknown environmental and physiological factors that are impossible to define or control in different patients [43, 44]. Human Organ Chips significantly reduce this problem by allowing a ground‐up, ‟synthetic biology″ approach to model and study complex organ‐level function by varying individual control parameters (e.g., cell types, chemical factors, mechanical cues, and other types of stimuli) independently and in defined combinations [45]. This novel ability to control all contributing factors enables matched comparative modeling of the same patient's cell and tissue responses before and after a critical stimulus, without the confounding factors of patient‐to‐patient variability, family history, medical history, and so forth [46, 47]. For example, one can use a Cervix Chip that is only comprised of one or two cell types (i.e., epithelium, stroma) to characterize the mucus composition profile due to production by only these human cell types in the absence of microbiome or immune cells, which is impossible in clinical samples. Then researchers can proceed with step‐by‐step addition of other cell types (i.e., endothelial, immune, nerve), hormones, microbiome, and so forth to identify how each factor contributes to or alters the overall biochemical and physical properties of the mucus layer. High‐resolution multi‐omics analysis combined with this versatile functional and structural characterization platform [12] can be used to tease out the precise mechanisms by which each variable, individually and in combination with other factors, modulates mucosal immunity. The resolved molecular profile of the mucosa and the underlying mechanisms that are unveiled will likely facilitate identification of new therapeutic targets as well as discovery of biomarkers that could predict mucosal health and disease susceptibility.

There is also currently a limited understanding of how host mucosal immunity influences a woman's susceptibility to pathogens and disease development, such as risk of sexually transmitted infections (STIs), dysbiosis, and chronic interstitial pelvic conditions. Improved understanding of the mechanisms involved will enable development of more effective therapeutics as well as prophylactics to enhance host protection, and thereby lower the risk and burden of sexual and reproductive diseases in women. But addressing these types of questions requires robust in vitro systems that can faithfully model the mucosa that lines the female reproductive tract. Microfluidic Cervix and Vagina Chips can be used to explore these problems because they are able to recapitulate both healthy physiology and pathophysiology associated with bacterial infections, while simultaneously enabling thorough characterization of the composition, structure, and function of the cervical mucosa, including its overlying mucus layer [12, 24]. A range of different disease conditions can be modeled on‐chip to study how an otherwise healthy mucosa responses to invasion by various infectious pathogens (viral and bacterial) as well as exposure to toxins, chemicals, or drugs. Furthermore, these conditions can also alter a healthy mucosa in a way that make it more prone to future insults [39, 48, 49], or make the host more susceptible to developing complex conditions, such as cancer, pelvic diseases, endometriosis, or pregnancy complications [39, 50, 51, 52, 53]. These are all largely understudied problems that can be addressed using human Organ Chip models of the female reproductive tract.

Host factors, including genetic background, ethnicity, pre‐existing conditions (i.e., diabetes, autoimmune disease), and hormonal states (menopause, pregnancy) are additional potential risk factors [32, 54, 55, 56, 57] that also can be mechanistically investigated using these preclinical models. For example, donor‐specific chips can be developed using primary cells isolated from particular donors or related donor groups to study and identify host‐specific factors that specifically affect the mucosal functions and immunity in that particular patient or population. For example, the Cervix Chip can be designed not only to replicate the normal cervical microenvironment but also to mimic genetic or disease‐associated alterations in mucus properties by either obtaining cells from patients with these disorders or using gene engineering approaches (e.g., CRISPR) to create these genetic changes. Thus, these preclinical human Organ Chip models of the female reproductive tract can help to enable more personalized approaches that can improve health disparities between different patient populations.

With the increasing recognition of the role of the microbiome in heath and disease states of the cervicovaginal niche [34, 58], it becomes increasingly important to answer the question of how commensal bacteria modulate host mucosal immunity. For instance, what are the underlying factors and processes by which the lower reproductive tract tolerates the commensal microbiome yet maintains sensitivity to detect and respond to pathogens without inducing harmful inflammation. Human Organ Chip models can be used to address these questions directly by designing host‐microbiome and host‐pathogen co‐culture studies. This should lead to a better understanding of the mechanisms that regulate the long‐term homeostasis of host‐microbiome interactions as well as how appropriate host responses are triggered during assaults by pathogens.

In the lower reproductive tract, innate as well as adaptive immune responses are thought to shape overall host immunity, but how these responses are controlled individually and in concert also remains unknown. Using the human Cervix Chip, we showed that in the absence of immune cells, the innate immune response of cervical epithelium can be modulated by the sex hormones, micromechanical cues, microenvironmental pH, and exposure to different bacterial communities [12]. The model can be further advanced by including immune cells, such as dendritic, macrophages, and T cells, to study how these adaptive immune components contribute to host immunity in the human cervix. Circulation of neutrophils in the endothelial lumen of human Organ Chip models have previously been shown to be critical for accurate modeling of, for example, bacterial infection and clearance as well as physiological host responses in vitro [59]. This highlights the importance of including immune cells in chip models for relevant recapitulation and studying disease pathophysiology and host immune responses. A Human Lymphoid Organ Chip [60] also has been developed that can form germinal center‐like lymphoid follicles with physiological responses to commercial vaccines, which potentially can be fluidically linked to the reproductive tract Organ Chip models to also study adaptive immune responses of the human host to microbiome and pathogens in vitro.

As new therapeutic interventions are emerging with the aim to enhance mucosal barrier function or modulate host immune responses (i.e., vaccines, hormonal treatments, and non‐antibiotic biotherapeutics) [61, 62, 63], their effectiveness and long‐term outcomes remain dependent on animal models that are often inaccurate predictors of human response as well as human clinical trials that are extremely expensive and time‐consuming. Human Organ Chip models provide an alternative yet robust platform for preclinical testing and optimization of new therapeutics before they proceed to clinical trials. Although Organ Chip models can be less expensive than animal models, their major advantage is that they provide more human‐relevant translational information, for example, about the safety and efficacy of new interventions before testing in clinical trials. Because drugs are introduced into the chips under dynamic flow, relevant drug exposure profiles (pharmacokinetic behavior) can also be modeled and studied on chips. Together, these features can facilitate the design of more effective human clinical trials and hence reduce the likelihood of failure in the late stages of drug development, saving millions of dollars in costs [64].

In addition to translational research, physiologically relevant Organ Chip models of the female reproductive tract also can advance understanding of the basic science of host mucosal tissues. For example, the endo‐, ecto‐, and transformation zones of cervix have been known for their distinct phenotype, function, and susceptibility to diseases such as neoplasia, HPV infection, and cervical cancer [65, 66, 67]. However, little is known about how each of these epithelial niches contribute to the physiology, pathophysiology, immunity and disease susceptibility of the reproductive tissues. Researchers could potentially model each region on‐chip to better understand the important physiological, molecular, and functional differences between these niches and how they contribute to overall host immunity or susceptibility to different pathogens and diseases.

### 1.3 Unveiling Mechanisms Underlying Host‐Microbiome and Host‐Pathogen Interactions

In the past, the female reproductive tract was thought to be sterile, and the gut was the only site where microbes were considered to reside in the human body in the absence of disease. The discovery of microbiomes in other parts of the human body, including the vagina, cervix, uterus, lung, and skin [68, 69] led to the realization that organ‐specific microbiome also plays a key role in health and in a plethora of diseases including those related to the female reproductive tract. For example, it is known that *Lactobacilli* bacteria species (*Lactobacillus* and particularly *L. crispatus*) dominate a healthy cervicovaginal microenvironment, and they are believed to play an important role in guarding against opportunistic pathogens and other infectious microorganisms [34, 70, 71, 72]. Many diseases such as aerobic vaginitis, bacterial vaginosis, candidiasis have been associated with a decrease in *Lactobacilli* in the vagina and an increase in opportunistic anaerobic microbes such as *Gardnerella vaginalis, Sneathia sp, Prevotella sp, Atopobium vaginae, Staphylococcus aureus, and Group B Streptococcus [* 37, 53, 73, 74, 75, 76, 77, 78, 79, 80, 81, 82, 83]. Importantly, clinical evidence shows that women with cervicovaginal dysbiosis are at higher risk of developing devastating conditions, including sexually transmitted diseases, pre‐term birth, infertility, and chronic inflammatory diseases [68, 81, 84, 85, 86]. Additionally, a *Lactobacillus*‐deficient microenvironment is the feeding ground for viral infections such as HPV, which can lead to an increased incidence of cervical cancer [51, 87].

The interplay between bacterial, fungal and viral communities is complex and plays an essential role in disease development. Current models to understand the interplay among these microbial communities are based on use of bioreactor culture systems, which are limited in clinical relevance because they fail to capture the role of the host in mediating these interactions. In addition to enabling study of multi‐microbial interactions in a human tissue‐ and organ‐relevant context, Organ Chip models can also be used to analyze host‐microbial interactions, and thus, provide better and more relevant insight into all the factors that contribute to disease development. To understand how alterations in the homeostatic balance contribute to disease development, it is first important to characterize how the microbiome influences the host and vice versa under healthy conditions. However, lack of appropriate models has slowed down research in this field. Conventional static culture models only support host‐microbiome co‐culture studies for up to 24 h before the bacteria take over the culture and kill the epithelial cells [88]. Importantly, the human Cervix Chip model overcomes these issues by producing a physiological mucus layer and allowing bacterial engraftment and stable co‐culture for 3 days or more [12]. Because the Cervix Chip utilizes primary human cells as opposed to cell lines, it also can be used to understand how various microbes affect normal human epithelial cell physiology and key host functions, including innate immunity as well as the production, composition, and physical properties of cervical mucus. Additionally, this model can be used to uncover molecular details related to infection mechanisms displayed by various pathogenic microbes. For example, little is known about the conditions and mechanisms employed by HPV to induce cervical cancer. Some reports suggest that hormone imbalances, cervicovaginal microbiome, or weakened immune system play a key role in the process by which HPV promotes cervical cancer formation [39, 51]. The Cervix Chip could be used to directly test these predictions and to tease apart the main drivers of HPV‐mediated cervical cancer induction. Furthermore, since these Organ Chips can give us an insight into host molecular responses by enabling analysis of the outflows of the epithelial and stromal channels individually, they provide a robust tool to identify biomarkers which may be secreted by the host in pre‐cancerous stages thereby helping to detect cervical cancers earlier than is possible today.

Additionally, cervicovaginal dysbiosis has been correlated with cervical cancer and with the increased risk of acquiring STIs, but the mechanisms underlying this phenomenon are still unknown. This too could be unveiled using the Cervix Chip model as dysbiotic bacteria have been shown to successfully engraft in both the Cervix and Vagina Chips [12, 24]. Using these models to co‐culture agents that cause sexually transmitted diseases (e.g., HIV, HSV, *Neisseria gonorrhea*, *Chlamydia trichomonas*) could help researchers better understand the role of cervicovaginal dysbiosis in these disorders [87, 89]. New insights into the underlying molecular mechanisms should enable development of drugs that target specific mediators of disease development that could be used to treat or prevent infections in the future.

The human Cervix Chip model can also provide researchers with a platform for preclinical testing of various drugs and probiotic therapies being developed. For example, Organ Chip models of liver, lung, kidney, bone marrow, and placenta have previously been used to test drug efficacies and toxicities [90, 91, 92, 93, 94, 95, 96]. For example, studies in which 27 different drugs with known hepatoxic and non‐toxic effects in animals and humans were tested in human Liver Chips revealed that this human‐relevant microfluidic culture model was significantly more accurate at predicting drug‐induced liver injury in humans than animal models [64]. A human Placenta Chip also has been used to study drug transport across the placental barrier to better understand if drugs administered to the mother can affect the fetus [95]. Similarly, the Cervix and Vagina Chips could be used to study how antibiotics or other therapies alter the cervicovaginal microbiome and host mucosa. Furthermore, some patients develop resistance to certain antibiotics, rendering the treatment ineffective, resulting in disease recurrence. To counteract this problem, Organ Chip models could be infected with bacteria released from cervicovaginal swabs from different patients, and various antibiotics or therapies could be tested to select personalized treatments that are more effective.

### 1.4 A Tool to Study the Role of the Cervical Mucosa in Fertility

Sperm must swim through the cervix and its mucosal secretions to reach the upper reproductive tract to fertilize the egg within the fallopian tube. Although a critical process during conception, knowledge of how the cervix, its mucus and changing properties impact the sperm function, embryo implantation, and the overall reproductive microenvironment remain poorly understood. To address these gaps, researchers have leveraged microfluidic in vitro models of the human cervix. One example employs a microfluidic chip that contains a hyaluronic acid gel to mimic cervical mucus without living cells that was used to quantify sperm locomotion in vitro, providing more physiologically relevant measurements of the sperm behavior [33]. However, the human Cervix Chip [12] contains human cervical tissues as well as mucus that is produced by those cells, and hence, it more closely recapitulates the human cervix than conventional 2D or 3D cultures. It may therefore be more useful for in vitro analysis of the effects of potential contraceptives on sperm motility and capacitation, as well as fertilization.

Although we know that the mucus composition varies during the menstrual cycle to support or inhibit sperm transport, the exact molecular mechanisms, especially the immune responses, microbiome interactions, and hormonal influences, remain inadequately explored. The human Cervix Chip can close this gap by providing a controlled physiological microenvironment where these complex interactions can be investigated in detail. Sperm can be directly introduced into the Cervix Chip to monitor their migration through the cervical mucus microenvironment. For example, a recent study demonstrated that when the Cervix Chip is infected with *Gardnerella vaginalis—*mimicking a bacterial vaginosis phenotype—it induces a significant decrease in the sperm motility associated with reduced mucus thickness and elevated inflammatory cytokines [97].

Cervical mucus also plays a fundamental role in fertility by supporting sperm survival, facilitating its transport, and protecting the upper reproductive tract environment from pathogens. This dynamic environment influenced by hormonal changes adjusts to create favorable conditions for conception. During ovulation, high levels of estrogen make the mucus watery (physically less restrictive to sperm) and alkaline to channel the sperm toward the uterus enhancing its motility and viability for fertilization [98, 99]. Outside this fertile period, a higher level of progesterone causes the mucus to thicken and become acidic, selectively forming a barrier that limits sperm entry, particularly filtering out lower‐quality sperm with compromised structure or motility [100]. The cervical mucus's ability to control sperm entry and movement plays a crucial role in maximizing fertilization success since the mucus filters, nourishes, and stores sperm to align with ovulation timing [101]. For this reason, tracking changes in cervical mucus is used as an ovulation predictor, with the texture becoming clear, slippery, and stretchy at the peak of the fertility. This change is so distinct that 93% of women can recognize and use these indicators to track ovulation [102, 103]. This predictable pattern allows for natural family planning methods.

Conversely, hormonal contraceptives, which increase progesterone levels, cause cervical mucus to thicken and maintain a barrier that blocks the sperm from entering the uterus effectively inhibiting fertilization by removing the typical fertile‐phase channels [98, 99, 100]. The conducive functions of the mucus also can be modulated by other factors affecting the overall fertility in women. Elevated levels of secretory leukocyte protease inhibitor (SLPI) may indicate an overactive immune response, which hinders sperm survival within the cervical microenvironment [104]. The Cervix Chip also can be designed not only to model the normal cervical microenvironment and its responses to external stimuli, but also to mimic genetic‐ or disease‐ associated alterations in mucus. For example, genetic mutations, such as DeltaF508 and R117H/7T commonly associated with cystic fibrosis, have been reported to alter the composition of cervical mucus making it less conducive to sperm movement, thereby reducing fertility potential [105]. Again, this could be modeled on‐chip by obtaining cells from patients with these conditions or inserting these mutations into healthy cells using genetic engineering approaches.

Infertility impacts 186 million people worldwide, with a quarter of cases remaining unexplained [106, 107]. Key interactions within the reproductive tract's mucosal epithelium, microbiota, and microenvironment are crucial to reproductive health, as studies indicate that infertile women often exhibit higher bacterial diversity [108] and reduced dominance of *Lactobacilli* species [109], with dysbiosis being more common in the lower reproductive tract [110, 111]. Despite these associations, microbial screening or treatment for unexplained infertility is not recommended due to the lack of confirmed causation [112]. This challenge is largely due to existing research tools that fail to replicate the complex interactions between male sperm and the female reproductive system. The Cervix Chip offers a unique way to study how changes in mucosal, microbial, and environmental factors affect sperm function, and hence, its use could facilitate the development of new treatments for infertility. Additionally, by using primary patient‐derived cells, the model can enable a personalized approach to identify causes and to customize treatments for infertile couples.

Beyond infertility, access to effective contraceptives is crucial for ensuring reproductive autonomy, and the Cervix Chip represents a promising platform for developing safer, non‐hormonal, and more effective contraceptive strategies. By faithfully replicating the structural and secretory environment of the human cervix, microfluidic in vitro models of the cervix permit direct evaluation of how various contraceptive modalities (e.g., spermicidal formulations, localized physical barriers) affect sperm motility and viability under more physiologically relevant conditions. Such microfluidic platforms have been promoted by the US Food and Drug Administration guidelines for use in comprehensive preclinical drug screening [113]. Consequently, the Cervix Chip may not only facilitate assessments of efficacy, safety, and cytotoxicity for both established and emerging contraceptive products, but also help to establish benchmarks for their optimal use, thereby informing the design of personalized contraceptive solutions.

### 1.5 Advancing Pregnancy Health

The cervix plays a crucial role in maintaining a healthy pregnancy by functioning as both a mechanical and biochemical barrier. Throughout pregnancy, it remains tightly closed, sealed by a mucus plug, and only physically dilates during initiation of labor to allow the passage of the fetal head. Additionally, the cervical plug is rich in antimicrobial peptides [114], which serve as a biochemical defense against pathogens. Despite these protective mechanisms, the cervix is not impervious to microbial invasion. When microbes breach this barrier, they can ascend to the uterine cavity, leading to adverse pregnancy outcomes, such as miscarriage, preterm labor, or stillbirth. A vaginal microbiome depleted of *L. crispatus* has been shown to be associated with a short cervix and an increased risk of preterm labor [115]. Similarly, the presence of *Ureaplasma parvum* in the cervical microbiome has been linked to chorioamnionitis and preterm birth, posing a threat to fetal health [116].

Although microbial infections account for 40% of preterm births [117], our understanding of how these pathogens interact with the cervix during pregnancy remains limited. Fundamental questions persist, such as why different women have distinct microbiome compositions and why the same microbes can trigger inflammation and preterm labor in some women while causing asymptomatic infections in others. Addressing these questions in human patients is challenging, but Organ Chip models of the female reproductive tract now offer an opportunity to address these pregnancy related questions directly. These chip models not only mimic the physical and biochemical microenvironment of the cervix but also allow researchers to investigate host responses under controlled conditions. For instance, a different human Cervix Chip has revealed that colonization by *U. parvum* [28] results in suppression of immunogenicity and that inflammation‐associated factors, such as lipopolysaccharide endotoxin (LPS) and TNFα alter the cervical phenotype [27]. Furthermore, integrating different types of analytical techniques with this technology, such as in‐line sensors that are directly integrated into the microfluidic chip [118, 119] for quantifying changes in pH, O_2_, barrier function, and metabolic activity as well as off‐line analyses (e.g., proteomics, transcriptomics, metabolomics, glycomics) can help researchers identify protective host factors and biomarkers for early detection, as well as stratify individuals at risk.

The Cervix Chip models also have the potential to improve therapeutic strategies for preventing premature cervical shortening and labor. Existing treatments, developed in the 1960s [120]^,^ are largely ineffective, and progress in translating new therapeutic targets to clinical practice has been slow. One significant barrier has been differences in labor mechanisms between animals and humans, along with inconsistencies in experimental design across studies. Standardized human Cervix Chip models could serve as valuable testbeds for evaluating promising treatments, including antibiotics and anti‐inflammatory drugs. For example, these chips could help elucidate why metronidazole alone fails to prevent preterm birth despite effectively treating bacterial vaginosis [121] and why inhibiting inflammation does not prevent *Group B Streptococcus* from ascending to the fetus. Moreover, human Cervix Chips can be leveraged to explore combinatorial therapies that simultaneously reduce bacterial load and mitigate inflammation, as well as investigate the potential anti‐inflammatory effects of small molecule drugs, biologics, and live biotherapeutic products, such as *L. acidophilus* [122].

A promising avenue for advancing research on preterm labor is the integration of multiple tissues (e.g., fetal membranes [123]) within an Organ Chip or fluidic coupling of multiple Organ Chips relevant to this condition within a single microfluidic platform. It would, for example, be interesting to investigate how different stimuli associated with preterm labor differentially trigger cervical shortening or premature rupture of fetal membranes [124] and myometrial contractions. Multi‐Organ Chip platforms can provide organ‐ and system‐level insights that go beyond the capabilities of individual chip models. For example, a microphysiological culture platform that integrates vaginal epithelial cells, ectocervix, transformation zone, endocervix, cervical stromal cells, and decidua cells, has been described that enables study of ascending infections [28]. Future models could also incorporate relevant hormonal and physical stimuli, such as mechanical deformations that the living cervix experiences, to more comprehensively replicate the processes involved in preterm birth. However, achieving this level of complexity is not without challenges. Combining primary cells from multiple distinct organs in a single device or single microfluidic multi‐organ platform, while ensuring their full differentiation and maintaining their native phenotypes, is difficult. The future success of this field will require advancements in both fundamental understanding of epithelial and stromal biology and development of more effective cell culture protocols, as well as innovative engineering solutions for complex and integrated Multi‐Organ Chip platforms.

### 1.6 Conclusions and Future Perspectives

The human cervical mucosa serves as a critical barrier for reproductive health, yet there is still much to learn about its composition and function, including the molecular and cellular mechanisms that underlie cervical immunity, diseases, and interactions with bacteria and pathogens. We also still do not understand how the physiological state of the cervix contributes to the success or failure of fertility and pregnancy. Advancements in microfluidic Organ Chip technology and its application to the female reproductive tract, as seen in the human Cervix and Vagina Chips reviewed here, offer transformative tools to model the complexity of the cervicovaginal microenvironment. These models enable stepwise exploration of mucosal immunity, host‐microbiome interactions, and responses to pathogens, toxins, and therapeutics. They also can facilitate investigations into the molecular drivers of infections, helping to identify new targets for the development of more effective drugs as well as biomarkers for early disease diagnosis. Beyond identifying therapeutic targets, the Cervix Chip offers a platform for comparing the efficacy of systemic versus topical drug delivery and evaluating the biocompatibility of novel biomaterials and ointments for localized treatment because compounds can be separately added to either the epithelial or stromal compartments [125].

The Cervix Chip platform also provides an innovative approach for studying fertility by simulating the host mucosa microenvironment and its impact on sperm function. This could provide new mechanistic insights into how the female cervicovaginal mucosa promotes or inhibits conception, in addition to enabling translational contraceptive research. Experimental research on pregnant individuals has historically been difficult limiting our understanding of female biology and pathophysiology during this transient but critical window of reproduction. The human Cervix Chip represents a promising tool for both basic and translational research in this area, which can enable development of improved interventions to prevent or treat pregnancy complications and preterm birth. These novel Organ Chip models of the female reproductive tract can also address issues relating to patient‐to‐patient variability in research by integrating donor‐specific cells and thereby advance personalized medicine strategies. Importantly, preclinical testing of emerging therapies can be facilitated by human Organ Chips and thereby, reduce reliance on animal models and increase success in costly clinical trials.

Despite the promise of Organ Chips, challenges remain. The biggest challenge is obtaining high‐quality human cells, along with relevant patient histories. Thus, there is a great need for human cell biobanks with comprehensive metadata linking a patient's history to microbiome composition and other relevant data. Establishing long‐term partnerships between researchers and clinicians will be crucial for overcoming these obstacles. Currently, the relatively higher costs of Organ Chips compared to conventional in vitro models impose some limitations to their widespread use. However, these costs are comparable to or less than the costs of animal studies, which fail to recapitulate human‐relevant responses. Furthermore, the cost of Organ Chips should decrease significantly with economies of scale over time as this industry is still in its infancy. Overall, we hope that from this review of the field, it is clear that Cervix Chip models hold significant potential for academic and industrial researchers and innovators who are interested in women's health. This technology can be used to close critical knowledge gaps, drive therapeutic innovation, and improve health outcomes for the sexual and reproductive health of millions of women globally.

## Author Contributions

Z.I. and D.E.I. conceptualized the manuscript structure. Z.I., A.S., A.G., and O.G. contributed to writing the manuscript. Z.I. and D.E.I. reviewed, edited, and finalized the manuscript.

## Conflicts of Interest

D.E.I. holds equity in Emulate, chairs its scientific advisory board, and is a member of its board of directors. The other authors declare no conflicts of interest.

## Data Availability

Data sharing is not applicable to this article as no new data were presented in this article.
